# Real-Time Gas Identification by Analyzing the Transient Response of Capillary-Attached Conductive Gas Sensor

**DOI:** 10.3390/s100605359

**Published:** 2010-05-28

**Authors:** Behzad Bahraminejad, Shahnor Basri, Maryam Isa, Zarida Hambli

**Affiliations:** 1 Institute of Advanced Technology, University Putra Malaysia, 43400 Serdang, Selangor, Malaysia; E-Mail: bbahraminejad@ieee.org; 2 Faculty of Engineering, University Putra Malaysia, 43400 Serdang, Selangor, Malaysia; E-Mail: misa@eng.upm.edu.my; 3 Faculty of Medical Science, University Putra Malaysia, 43400 Serdang, Selangor, Malaysia; E-Mail: zarida@medic.upm.edu.my

**Keywords:** gas sensor, transient response, electronic nose, feature extraction

## Abstract

In this study, the ability of the Capillary-attached conductive gas sensor (CGS) in real-time gas identification was investigated. The structure of the prototype fabricated CGS is presented. Portions were selected from the beginning of the CGS transient response including the first 11 samples to the first 100 samples. Different feature extraction and classification methods were applied on the selected portions. Validation of methods was evaluated to study the ability of an early portion of the CGS transient response in target gas (TG) identification. Experimental results proved that applying extracted features from an early part of the CGS transient response along with a classifier can distinguish short-chain alcohols from each other perfectly. Decreasing time of exposition in the interaction between target gas and sensing element improved the reliability of the sensor. Classification rate was also improved and time of identification was decreased. Moreover, the results indicated the optimum interval of the early transient response of the CGS for selecting portions to achieve the best classification rates.

## 1. Introduction

Among different types of chemical gas sensors, metal oxide semiconductor (MOS) gas sensors are widely available and used in fabrication of gas monitoring and artificial olfactory systems [1,2]. As with other chemical gas sensors, the MOS group generally generates the output response based on trace of target gas (TG) in reaction to their sensing element, which, in turn, generates the output in terms of electrical conductivity variation. This trace generally depends on nature and concentration of the TG in a complicated way, which makes fabrication of a simple selective gas sensor difficult [3–5]. Most of the reported efforts to fabricate a selective MOS gas sensor have generally focused on material modification and optimization. Applying a catalyst on the effective surface of MOS [6–10], additive material [11–19], or a combination of these methods [20] can affect the sensitivity and partially optimize the selectivity of the sensor. Variation of thickness of sensitive film can also enhance partial selectivity of sensors [21,22].

Besides mentioned studies on MOS gas sensors, so many other researches on different types of chemical gas sensors have been reported to achieve a perfect selective gas sensor. A major achievement of those efforts was to improve sensitivity and partial selectivity of chemical gas sensors. Fabrication of a single selective gas sensor, for use in a wide range of applications and detecting different TGs, is still difficult and most of the prototype products have been applied for hazard detection or monitoring level of contaminations.

Since sensitivity of MOS gas sensor to particular TG is directly related to the operating temperature of the sensor [23], modulated-temperature methods along with some analyzing approaches have also been applied to enhance the selectivity. Studies on a single gas sensor with temperature modulation have indicated the value of this selectivity even in complex gas mixtures [24–30]. Employing an array of chemical gas sensors with improved sensitivity and partial selectivity, such as above presented MOS gas sensors, in electronic nose (e-nose) technology has solved the selectivity problem in different applications. However, these methods (temperature modulation and e-nose) make the classification algorithm more complicated and some common problems occur similar to other methods.

These gas monitoring and identification systems have generally applied steady state response of chemical gas sensors, which results in long time exposition. Long time exposition of a sensor in the presence of a target gas can cause irreversible interactions between the TG molecules and the surface of sensing element. Therefore, recovery time can be increased, baseline value varies in time, repeatability and reliability of sensor are reduced, as are some effects present in the output of sensor such as drift [2]. Decreasing sensitivity of the sensor and increasing difficulties of classification methods can be mentioned as other effects. These problems may be accrued in either a single sensor or an array of sensors. An electronic nose system is also more complicated and costly than a single gas sensor which can be more suitable to employ in some simple processes. In other investigations, application of transient response for enhancing recognition abilities of the electronic nose have been presented [30–32]. Presented results have indicated improved classification. Although the transient response of gas sensors has been applied in these approaches, response of sensors has been recorded up to steady state level and long time exposition problems have remained unsolved. The complicacy of classification algorithms has also increased in mentioned studies.

Some other investigations have been carried out to minimize exposure of the sensor to the TG molecules and to assist extraction of original attributes of signal by keeping the sensor abilities. Researchers have attempted to focus more attention on the transient part of the sensor array output to capture effective features from this portion. Faster signal acquisition and slower aging process of sensors are other advantages of this method. This method has presented improved classification rates by applying part of transient response along with multilevel signal decomposition [33,34].

All above reviewed methods have investigated the improvement of the selectivity based on enhancement of sensor technology or signal processing techniques, but the trace of TG on sensing element of the gas sensor has been the only factor applied to generate output features. Some other approaches have employed new sensor structures that not only use the above mentioned factor, but also increase the selectivity by applying other identifying factors.

The analysis and useful gas sensing properties of a novel single gas sensor structure which was called capillary-attached gas sensor (CGS) have recently been investigated. Definition of transient response in the CGS is the physical process of a TG diffusion through a capillary tube [35]. Quantitative evaluations have stated that transient response of the CGS is robust against aging and environmental poisoning. Independence of the CGS normalized transient response to gas concentration has also been indicated analytically. It has also been stated that the information regarding the nature of a TG can easily be extracted from the corresponding transient response of the CGS and any specifically defined point on the transient responses can be applied to compare and detect different gases along with temporal analysis [35–37]. However, all previous studies on the CGS have also been investigated by temporal analysis of the normalized response of the CGS generated after recording the response of the CGS to different TGs up to steady state level. According to the CGS structure, transient time is longer than a simple MOS gas sensor which can intensify the long time exposition effects.

CGS transient response contains valuable detecting data related to diffusion of TGs. Therefore, the aim of this study is the assessment of real-time gas identification by applying selected portion from the beginning of the CGS transient response to achieve optimum classification and decrease the time of exposition as much as possible. Different selected portions of the CGS transient response are analyzed including the first 11 samples to the first 100 samples in order to study the effects of portion length and find the optimum length for portion selection. This approach not only decreases the time of exposition but also applies the diffusion parameter of a TG as an extra factor for identification. These advantages can result in improved sensor reproducibility by decreasing the interaction time of a TG and sensing element and decelerating the aging process. Therefore, high classification performance can be obtained by applying a simple classifier.

In this paper, results of the experimental work on detecting short-chain alcohols by a prototype CGS are reported. Diverse features were extracted from certain portions of the CGS transient responses. Principal component analysis (PCA) and linear discriminant analysis (LDA) were employed to extract features, as the most applied method for dimension reduction in the machine olfaction [38]. Quadratic classifier, multi layer perceptron (MLP) artificial neural network, k-nearest neighbors (k-NN), and support vector machine (SVM) classification methods are applied to complete the identification process. Finally, K-fold cross validation is used to evaluate classification results.

## 2. Experimental Method

### 2.1. CGS Prototype Structure

**Figure 1.**
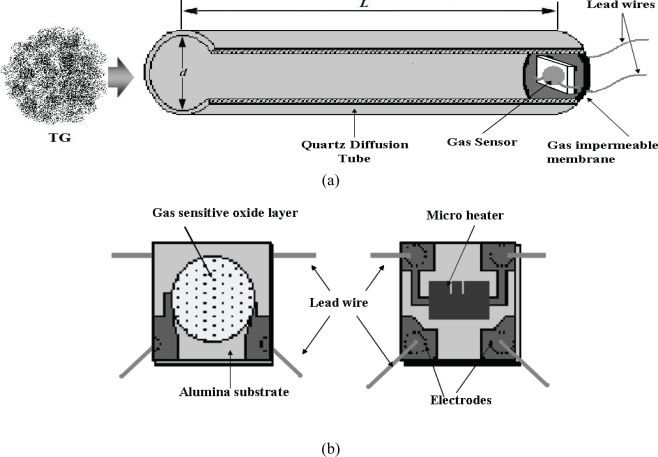
(a) Schematic of the prototype CGS fabricated. (b) Schematic of sensing element of the CGS.

The schematic diagram of the fabricated prototype CGS is shown in Figure 1. The device included a MOS conductive gas sensor as a chemical gas sensor and a glass tube. The tube was attached to the sensor in an airtight manner; therefore, diffusion through the effective length of the tube (*L* in Figure 1a) was the only path for a TG to affect the gas sensor. In the prior experimental work, commercially resistive gas sensors and quartz tubes with internal diameters larger than 7mm had been employed to fabricate a CGS prototype [35]. In the present work, a tin oxide gas sensor for general purposes and pure-air-filled quartz tube were used to fabricate the sensor. Schematic diagram of tin oxide gas sensor is presented in Figure 1b. Sensor structure included thick film layer of tin oxide on one side of an Alumina substrate and a Ruthenium oxide heater fabricated on the other side of the substrate. A quartz tube of 3 mm diameter and 55 mm length was selected to fabricate the CGS with optimum dimensions. These dimensions were selected to optimized selectivity, response time and recovery time.

Longer length and smaller diameter increase the selectivity but make the response time and recovery time longer. The negative effects of dimension on recovery time are more than the response time, such as the long-time recovery period in smaller diameters and longer lengths. Therefore, effects of long time exposition are increased. Sensitivity to low concentrations is also decreased by decreasing tube diameter [39].

For a simple MOS gas sensor, the output is the variation of sensing material conductivity in the presence of a TG (ΔG) and it is described as below [40,41]:

(1) $$\Delta G={\mathrm{SC}}^{m}$$

Where, *C* is the concentration of a TG and *S* and *m* are parameters related to characteristics of a sensor and nature of a TG. In the CGS structure, it is assumed that gas is equilibrated in the environment before measurement. A TG has to diffuse along the effective length of the diffusion tube (*L* in Figure 1a) before reaction with MOS gas sensor. This means concentration changes in time. Then, Equation (1) will be changed to the following format:

(2) $$\Delta G(t)=\mathrm{SC}{\left(t\right)}^{m}$$

Where *C(t)* is the concentration of a TG in the time *t* after beginning of diffusion at the close end of the CGS where the MOS sensor is located. The relation of *C(t)* to length and diffusion has been described previously, and it has been implied that TG with the higher diffusion coefficient generates the faster response [35].

### 2.2. Measurement System

**Figure 2.**
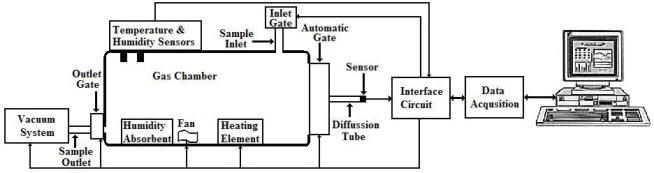
Schematic diagram of the measurement system designed.

Static method is used in the measurement processes, where volatiles have to be equilibrated in a sealed chamber before starting the measurement [32]. The schematic diagram of the measurement system which was applied in recording of the CGS responses is presented in Figure 2. The gas chamber had a volume of 20 liters and was made of glass. Prototype CGS was kept at operating conditions in clean air for at least 10 minutes before each measurement. Since interfering environment parameters such as temperature and relative humidity affect reproducibility of MOS gas sensor, humidity and temperature of the chamber were monitored continuously and kept at a constant level to eliminate interfering environmental factors and increase the reliability of the measurement system over time. CGS was attached to the gate of the chamber horizontally to eliminate gravity effects [35].

For each experiment, the automatic impermeable gate located at the open-end part of the prototype CGS was opened to the gas chamber at *t* = *0* and recording of the response was started synchronously. According to properties of the applied MOS gas sensor, a voltage divider circuit with DC power supply was applied to drive the gas sensor. Change of voltage in the constant resistor was used to measure the change of conductivity of the sensor. The output voltage was transferred to PC by a data acquisition system to calculate the variation of sensor conductivity (G_s_(k)). Sampling time was selected as one second. Real-time software designed for monitoring, recording and processing data was used.

During the recovery period, remaining polluting gases were diffused out from the chamber and diffusion tube by a vacuum pump to decrease the recovery time and eliminate long time exposition effects.

### 2.3. Preprocessing

**Figure 3.**
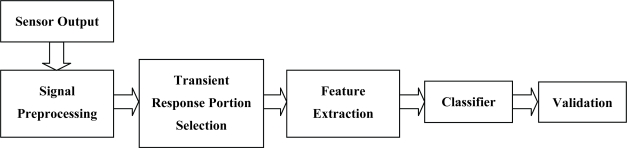
Flow diagram of processing method applied.

A flow diagram of the processing method is presented in Figure 3. Signal preprocessing must be applied to modify the sensor response and minimize the impact of disturbances, which is generated by unequal responses of sensor and variability due to environmental disturbances. Preprocessing methods can include any of these three major categories: baseline manipulation, compression, and normalization [31]. Baseline manipulation was applied to reduce the effect of sensor drift in this study. Sensor drift causes an unstable response over time with a slow and random variation of the baseline of the response generally. This manipulation based on the differential method was applied in subtracting each sampling by the initial baseline value of transient response [31,38,42]:

(3) $$\Delta G_{S}\;(k)=G_{S}\;(k)-G_{S}\;(0)$$

Where;

*G_s_* (*k*) is the original output of sensor.
*G_s_* (0) is the initial baseline output of sensor.
Δ*G_s_* (*k*) is the adjusted output value of sensor.

Manipulated transient response could be compressed by three different groups of methods: sub-sampling methods, parameter extraction methods and system identification methods, but according to intensive computation, sub-sampling and parameter extraction methods are generally more applied [43]. In accordance with the aim of this study, the sub-sampling method was employed and portions of raw samples of transient response after baseline manipulation were selected for assessment of classification performance based on original identification parameters of the signal. The optimum length of selected portions could be studied according to the nature of the transient response. To remove the probable limitation caused by applying raw samples, the measurement system was modified and experiments were done in a controlled environment.

### 2.4. Odor Database

To evaluate classification performance, four short-chain alcohols (Methanol, Ethanol, 2-Propanol and 1-Butanol) were applied to generate the responses. 12 different concentrations (50, 100, 150, 200, 300, 400, 500, 600, 700, 800, 900, 1,000 ppm) were selected for each TG. The experiment was repeated 5 times in different time intervals for each concentration to assess the reproducibility of the measurement system. Transient responses of 240 experiments were applied to generate the main database of responses. Summary of odor database is presented in Table 1.

### 2.5. Feature Extraction and Classification

Since the aim of this research was detecting target gases based on a selected portion of the early transient response, data sets were generated including the first 11 samples to the first 100 samples extracted from each baseline manipulated transient response. Each dataset contains extracted features of 240 experiments for all odors. Finally, 90 generated data sets were evaluated to find an optimum portion for classification. The kth data set can be defined as follows:

(4) $$\mathrm{DB}(k)=\left[\begin{array}{ccc} \Delta G_{L_{1},1}^{1}\;(0) & \cdots & \Delta G_{L_{1},1}^{1}\;(k)\\ \begin{array}{l} \vdots \\ \Delta G_{L_{i},r}^{c}\;(0)\\ \vdots \end{array} & \ddots & \begin{array}{l} \vdots \\ \Delta G_{L_{i},r}^{c}\;(k)\\ \vdots \end{array}\\ \Delta G_{L_{12},5}^{4}\;(0) & \cdots & \Delta G_{L_{12},5}^{4}\;(k) \end{array}\right]$$

Where;

*k* = 11, 12, 13, … 100; indicates the number of samples in the selected portion of transient response.
*c = 1, 2, 3, 4;* is the class number.
*L_i_* is the concentration level where;
*L* = [50, 100, 150, 200, 300, 400, 500, 600, 700, 800, 900, 1,000] and
*i* = 1, 2, ..., 12, and
*r* = 1, 2, …, 5; is the repetition number for different experiments of the same concentration in time.

Diverse extracted features from the steady state and transient response of the sensors have been applied in gas identification such as relative, log parameter, difference, fractional, derivative, Fourier coefficient, integration, and wavelet coefficient [42,44,45]. In this study the main features were extracted from transient responses after baseline manipulation. Gradient of selected features and fast Fourier transform (FFT) coefficients of selected data sets were also employed to compare different feature data sets in detection performance.

Feature reduction methods are widely used to eliminate the curse of dimensionality in classification and improve efficiency, classification performance and ease of interpretation and modeling [46,47]. Among different types of feature reduction methods, principal component analysis (PCA) and linear discriminant analysis (LDA) are widely used in gas identification system and machine olfaction [38,43]. PCA is a kind of signal representation method that chooses maximum variance directions to make the projection. These directions are defined by the first eigenvectors corresponding to the largest eigenvalues of the covariance matrix of input data; where covariance of input data is:

(5) $$\sum_{G}=E[{\left(G-\mu \right)}^{T}(G-\mu )]$$

LDA is a signal classification method to minimize class separability in a direct way and make samples from compact bunches and the different bunches far from each other. First eigenvectors of the multiplication result within class covariance inverse matrix and between class covariance matrices are applied to make projections. It can also define as a linear projection (*W*) that makes following objective function maximum [31,38,48,49]:

(6) $$J(W)=\frac{W^{T}\;\sum_{B}\;W}{W^{T}\;\sum_{W}\;W}$$

Where;

*∑_B_* is between class covariance matrix, and
*∑_W_* is within class covariance matrix.

Generally, PCA and LDA are applied widely along with different classifiers to assess the ability of the gas identification system. Extracted features by PCA are also projected to present the separability of different classes. Projections of extracted features by LDA are also applied to study the compactness of features from the same class and variance of the features from different classes [38,43]. In this study, both PCA and LDA methods were applied to extract features for all 90 data sets. Extracted features by PCA and LDA were applied for gas classification by employing different classifiers to investigate the abilities of early portions of transient response in fast gas identification.

Quadratic classifier, multi layer perceptron (MLP) classifier and k-nearest neighbor (k-NN) classifier are the most well-known methods in the area of gas classification [38,43]. SVM classifier has also been reported to have a high classification rate in gas identification [50–52]. The quadratic classifier is the simplest approach to approximate the largest posterior probability by assuming that for each class likelihood function is unimodal Gaussian density, and generate quadratic hyper surfaces as decision boundaries between classes [38,48]. MLP is the feed-forward neural network which includes simple processing elements or neurons, and results in complex nonlinear regression. This regression can be trained by adjusting weights of elements of the network, using a gradient descent method, which is called back propagation (BP) of errors [38,47,53]. K-NN selects the nearest k samples in the database to the unlabeled data and chooses the class with maximum members between its k-nearest selected neighbors [31,48,49]. Support vector machines (SVMs) are a kind of related supervised learning technique employed for classification. If every member of a given set of training examples belongs to one of two classes, an SVM training algorithm generates a model that predicts whether a new testing example categorized to one class or the other [50,52]. Extracted features by PCA were classified with the quadratic, MLP, k-NN, and SVM classifiers were selected as favored classifiers in this study.

**Figure 4.**
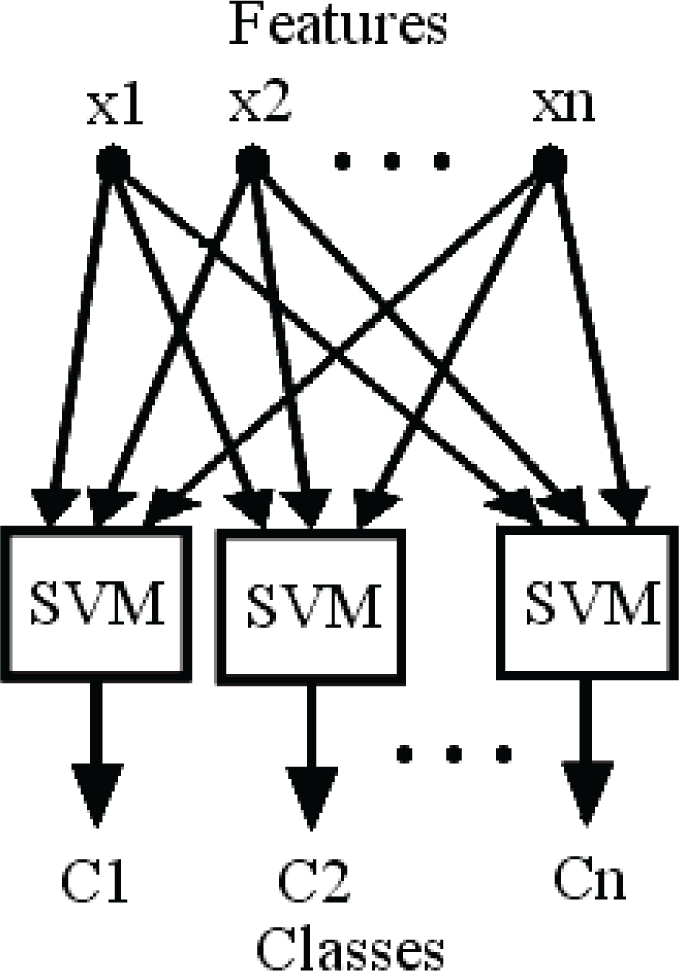
Structure of the SVM classifier applied.

A feed forward neural network was used as MLP classifier to evaluate the classification of the extracted features [47–49,53]. 3-7-4 layer structure (3 inputs, 7 neurons in one hidden layer and 4 outputs) was selected for neural network with the Levenberg-Marquardt (LM) optimization algorithm [42]. The number of nearest neighbors (k) for the k-NN classifier was selected by trial and error, which provided the highest classification rate. Distances were calculated based on Euclidean distance for this classifier. The presented structure in Figure 4 was applied as an extended SVM classifier in this study [54].

Finally, K-fold cross validation was used to calculate the classification rates as a well-known estimation of prediction error [38,49,55]. This method divides data set into K subsets. K-1 subsets are used as the training data set. The remaining subset is used as the testing data set. Average value across K trials of the testing data sets, indicates classification rate. Five-fold cross validation (k = 5) was used in this study to calculate the classification rates of each approach.

## 3. Result and Discussion

**Figure 5.**
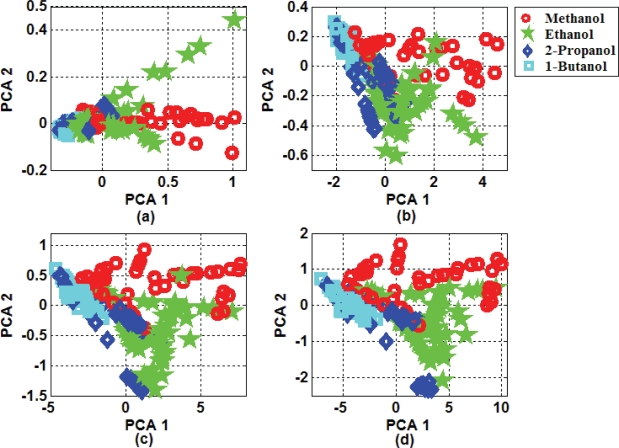
(a) PCA Projection results of the first 25 samples selected portions of transient response. (b) The same for the first 50 samples. (c) The same for the first 75 samples. (d) The same for the first 100 samples.

Two-dimensional projections of extracted features from baseline manipulated transient responses by PCA for selected portions including the first 25, 50, 75 and 100 samples of transient response are presented in Figure 5. According to presented results, extracted features by PCA indicated acceptable separability. Overlaps between classes decreased by increasing number of samples in selected portions. Then, better classification performance should be achieved.

**Figure 6.**
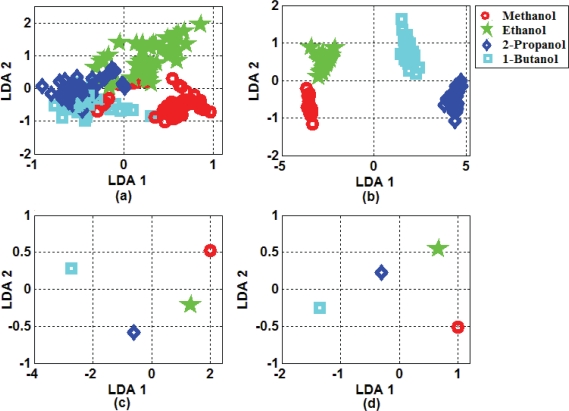
(a) LDA Projection results of the first 25 samples selected portions of transient response. (b) The same for the first 50 samples. (c) The same for the first 75 samples. (d) The same for the first 100 samples.

Two-dimensional projections of extracted features by LDA including the same selected portions are shown in Figure 6. For the selected portion including 25 samples, overlap between some classes was presented, and classification performance could be affected. Other illustrated portions presented high separability between classes. Projected features also indicated high compaction within classes. Compactness for selected portions including more than 50 samples was perfect. Presented results based on extracted features by LDA predict low error classification performance for selected portions of the transient response.

According to selected feature reduction and classification methods, eight different approaches were evaluated including classification of extracted features by PCA and LDA based on four classifiers. Testing samples of each data set were projected to three dimensions by applying projection vectors, which were generated by PCA and LDA from training samples. Then, they were applied to classifiers either including data of training samples or trained by training samples.

**Figure 7.**
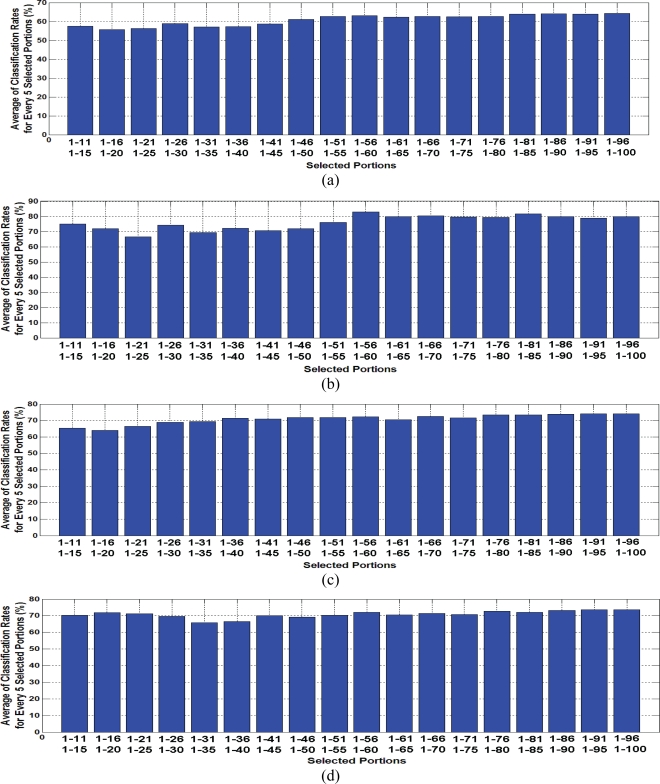
(a) Averages of evaluated classification rates for extracted features by PCA classified by the quadrate classifier. (b) The same for k-NN classifier. (c) The same for MLP classifier. (d) The same for SVM classifier.

**Figure 8.**
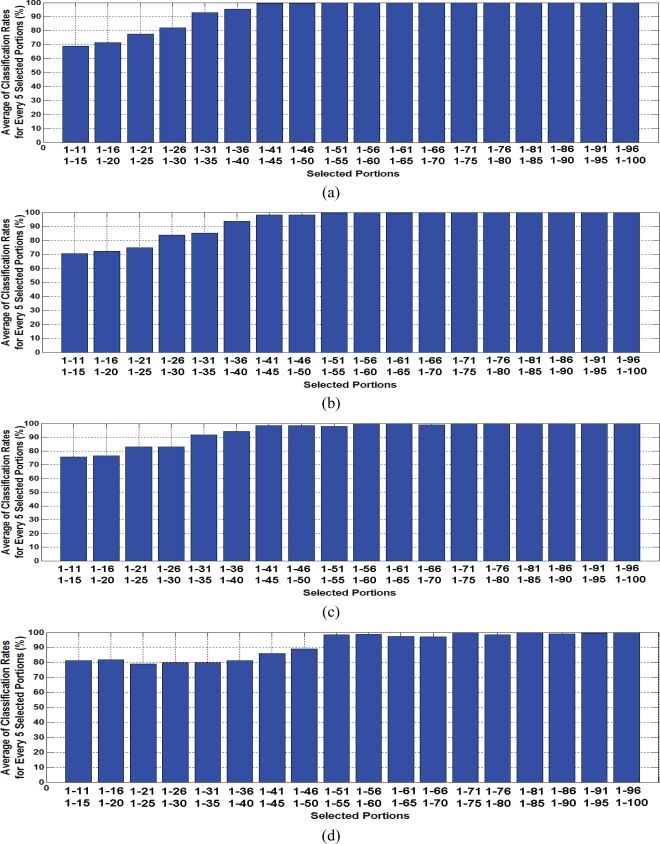
(a) Averages of evaluated classification rates for extracted features by LDA classified by the quadrate classifier and (b) The same for k-NN classifier. (c) The same for MLP classifier. (d) The same for SVM classifier.

Averages of evaluated classification rates based on extracted features by PCA and LDA from baseline manipulated transient responses have been summarized in Figure 7 and 8, respectively. Details of classification rates are also presented in Table 2. Each bar in presented figures indicates the average of evaluated classification rates for every 5 continuous selected portions of the CGS transient response. For example, the first bar presents the average of evaluated classification rates for selected portions including the first 11, 12, 13, 14 and 15 samples of recorded transient responses. Evaluated results for PCA-quadratics are presented in Figure 7a. Results indicated about 60% classification rate based on early part of the CGS transient response, especially for selected portions including the first 46 samples and above. The PCA-kNN approach presented similar results in comparison with the previous method in shape (Figure 7b), but classification rates increased about 20% for every selected portion. However, the second half of the selected portions, including more than the first 50 samples of transient responses, indicated a better classification of around 80%.

Presented results of PCA-MLP in Figure 7c indicated classification rates with an accuracy rate of about 70%. For SVM classifier, the general shape of classification rates was saved, but variation of evaluation results is smoother than other classifiers and the rate is around 70%.

For extracted features by PCA, k-NN classifier performed the best classification. General review of evaluated classification performances approximately indicated the flat rate of classification for all selected portions. However, the maximum classification was about 83%, and it resulted from selected portions including the first 55 to 60 samples of transient responses. The results imply the ability of the early selected portion of transient response of the CGS for gas identification.

Evaluated classification results based on LDA feature extraction as a supervised method, states different classification rates (Figure 8). The lowest classification rate of about 70% happened in early transient response portions. For selected portions including more than the first 40 samples, results presented perfect classification performance of up to 100%. All four classifiers performed similarly. However, the quadratic and MLP classifiers achieved more than 90% faster in classification performance. This means that high classification accuracy is possible by applying portions including the first 30 samples of the transient response.

Comparing details of classification performances in four approaches implied that the least number of samples for perfect classification was presented by the quadratic classifier results. SVM illustrated better classification rates for early selected portions with less than 25 samples. However, it was the latest approach in achieving over 90% in classification accuracy.

The evaluation of classification performance of extracted features based on the gradient of transient responses and FFT coefficients of transient responses is illustrated in Table 3 and 4, respectively. The evaluation results based on gradient method presented closed results to baseline manipulation. However, baseline manipulation had a general advantage. The results based on FFT technique presented the lowest classification rates in comparison with above methods. It indicated the relation of the CGS response to the temporal characteristics of the CGS response.

A general overview to all applied methods, confirms that the classification performance was related directly to the nature of transient response of the CGS. Therefore, generated features from original signals after the preprocessing step are the most suitable features for gas identification. All evaluated classification techniques have approximately generated a similar shape over the complete selected interval for study. Variation of classification rates was basically due to applying different feature extraction methods. LDA as a supervised method generated better features than PCA as an unsupervised method.

The presented results proved that an early selected portion of the CGS transient response included identification data for perfect classification. The optimum portions for classification must be included in about the first 45 samples of the beginning of the CGS transient response. Therefore, early selected portion of the CGS transient response can be applied for achieving high classification rate in real-time gas identification as a single sensor system or a sensor array. It can also minimize problems of long time exposition of sensing material in MOS gas sensors.

## 4. Conclusion

The fabrication of a selective gas sensor with distinct transient responses to TGs of different natures was described. The device had a simple structure including MOS conductive gas sensor attached to the glass tube. An experimental evaluation of the device indicated that the process of TG diffusion through the capillary tube, in comparison with the process of detection by the sensor element, configured its transient response to a prevailing TG. Ability of the Capillary-attached conductive gas sensor in real-time gas identification was investigated. Selected portions of the CGS transient response were applied to generate diverse features for gas classification. Combination of PCA and LDA feature extraction methods with the quadratic, k-NN, MLP and SVM classification were applied as classifier systems.

The algorithm was practically implemented and the ability of the designed system to distinguish between four different combustible target gases was demonstrated. Reported results in this paper indicate high classification rates based on extracted features from the early part of the CGS transient response for real-time gas identification. Reported ability of the CGS structure can apply to fabricate a real-time single sensor or a sensor array for fast gas identification and reduce the problems due to long time exposition of sensing material in chemical gas sensors.

## Figures and Tables

**Table 1.** Detail of the data sets and classes generated.

Class label	Alcohol	No. of samples in each concentration	No. of total samples	No. of samplings per sample
1	Methanol	5	60	100
2	Ethanol	5	60	100
3	2-Propanol	5	60	100
4	1-Butanol	5	60	100

**Table 2.** Averages of evaluated classification rates for extracted features from baseline manipulated responses.

Length of Sampled Feature	Feature Reduction Technique							
	PCA				LDA			
	Classifier				Classifier			
	Quadratic	KNN	ANN	SVM	Quadratic	KNN	ANN	SVM
11–15	57.55	75.00	65.40	70.26	68.88	70.50	75.59	81.20
16–20	55.61	72.00	63.96	71.80	71.43	72.30	76.62	81.83
21–25	56.30	66.49	66.43	71.30	77.55	74.90	82.99	79.10
26–30	58.88	74.20	68.84	69.64	82.14	83.88	82.95	79.93
31–35	57.04	69.43	69.31	65.92	92.86	85.31	91.75	79.76
36–40	57.35	72.24	71.33	66.53	95.41	93.67	94.27	81.27
41–45	58.78	70.53	70.83	70.03	99.49	98.27	98.36	85.80
46–50	61.12	71.88	71.71	69.13	99.49	98.27	98.47	88.87
51–55	62.65	76.04	71.87	70.35	100.00	99.49	98.00	98.50
56–60	63.06	82.90	72.17	72.06	100.00	100.00	100.00	98.83
61–65	62.40	79.96	70.39	70.45	100.00	99.59	99.90	97.30
66–70	62.65	80.50	72.56	71.31	100.00	100.00	99.08	97.06
71–75	62.45	79.59	71.51	70.75	100.00	100.00	99.90	100.00
76–80	62.65	79.47	73.32	72.70	100.00	100.00	100.00	98.60
81–85	63.98	81.67	73.36	72.08	100.00	100.00	99.80	100.00
86–90	64.20	79.84	73.80	73.16	100.00	100.00	99.90	99.09
91–95	63.90	78.73	73.97	73.56	100.00	100.00	100.00	99.50
96–100	64.40	79.96	74.00	73.70	100.00	100.00	100.00	100.00

**Table 3.** Averages of evaluated classification rates for extracted features by gradient method.

Length of Sampled Feature	Feature Reduction Technique							
	PCA				LDA			
	Classifier				Classifier			
	Quadratic	KNN	ANN	SVM	Quadratic	KNN	ANN	SVM
11–15	55.31	63.78	66.73	71.30	73.67	63.67	73.98	77.80
16–20	57.14	60.71	67.30	72.10	74.39	72.65	74.92	76.25
21–25	58.98	60.50	62.66	72.04	79.18	80.71	80.94	75.66
26–30	58.98	64.60	66.85	71.34	86.53	88.98	85.08	79.86
31–35	57.86	65.61	70.97	70.80	94.90	93.27	91.91	79.25
36–40	59.08	64.69	70.39	72.23	98.57	97.45	95.60	80.95
41–45	58.57	65.70	69.66	71.65	99.49	99.90	99.18	86.05
46–50	59.69	66.12	71.70	73.18	99.10	99.39	98.98	90.50
51–55	61.12	70.51	72.10	71.70	98.98	100.00	99.20	97.18
56–60	62.45	72.30	69.74	70.43	100.00	100.00	99.90	100.00
61–65	62.04	72.55	70.68	69.52	100.00	100.00	100.00	100.00
66–70	60.82	71.02	72.17	71.90	100.00	100.00	100.00	100.00
71–75	61.00	74.29	72.80	71.99	100.00	100.00	100.00	99.50
76–80	60.71	72.14	72.30	72.08	100.00	99.30	99.69	100.00
81–85	62.14	74.49	72.80	73.21	100.00	99.69	99.80	99.10
86–90	63.88	75.92	72.58	72.66	100.00	100.00	100.00	99.22
91–95	65.00	73.98	71.22	74.06	100.00	100.00	99.70	100.00
96–100	65.00	75.20	74.24	72.07	100.00	100.00	100.00	99.02

**Table 4.** Averages of evaluated classification rates for extracted features by FFT method.

Length of Sampled Feature	Feature Reduction Technique							
	PCA				LDA			
	Classifier				Classifier			
	Quadratic	KNN	ANN	SVM	Quadratic	KNN	ANN	SVM
11–15	35.41	24.80	34.47	72.30	34.39	50.92	57.17	65.17
16–20	35.82	20.00	33.40	73.40	35.51	53.47	62.40	68.30
21–25	36.12	19.10	32.50	73.10	36.73	63.47	61.35	63.35
26–30	34.30	20.30	36.00	68.50	41.40	67.96	73.69	64.71
31–35	34.39	21.40	35.86	71.20	40.61	71.53	73.29	64.29
36–40	35.71	18.78	36.79	70.30	35.71	87.04	90.05	68.84
41–45	34.49	18.27	35.98	69.93	34.80	94.69	89.69	63.27
46–50	35.92	20.92	35.10	71.77	34.20	89.59	93.57	65.90
51–55	36.43	22.45	33.97	72.61	24.90	71.22	58.78	67.61
56–60	36.53	22.96	32.61	71.83	25.71	66.12	58.50	64.19
61–65	34.18	25.41	34.29	73.79	23.98	65.10	56.70	70.00
66–70	32.45	25.00	37.56	72.35	23.16	67.04	56.11	74.90
71–75	30.31	24.29	38.50	71.59	27.04	66.84	48.88	74.60
76–80	29.39	23.16	39.03	72.34	21.12	58.98	47.26	78.21
81–85	28.98	21.73	42.09	71.33	26.02	61.94	46.94	87.12
86–90	27.96	17.65	42.59	72.53	26.12	57.24	52.48	85.21
91–95	28.37	16.33	42.75	71.19	22.96	57.86	54.14	84.89
96–100	28.37	13.57	42.80	72.33	22.24	55.31	54.90	87.30

## References

[1] Nanto H., Stetter J.R., Pearce T.C., Schiffman S.S., Nagle H.T., Gardner J.W. (2002). Introduction to chemosensors. Handbook of Machine Olfaction: Electronic Nose Technology.

[2] Arshak K., Moore E., Lyons G.M., Harris J., Clifford S. (2004). A review of gas sensors employed in electronic nose applications. Sens. Rev.

[3] Nakata S., Ojima N. (1999). Detection of a sample gas in the presence of an interferant gas based on a nonlinear dynamic response. Sens. Actuat. B.

[4] Nakata S., Akakabe S., Nakasuji M., Yoshikawa K. (1996). Gas sensing based on a nonlinear response: Discrimination between hydrocarbons and quantification of individual components in a gas mixture. Anal. Chem.

[5] Fort A., MacHetti N., Rocchi S., Serrano B., Tondi L., Ulivieri N., Vignoli V., Sberveglieri G. (2003). Tin oxide gas sensing: Comparison among different measurement techniques for gas mixture classification. IEEE Trans. Instrum. Meas.

[6] Schweizer-Berberich M., Zheng J.G., Weimar U., Göpel W., Bârsan N., Pentia E., Tomescu A. (1996). The effect of Pt and Pd surface doping on the response of nanocrystalline tin dioxide gas sensors to CO. Sens. Actuat. B.

[7] Williams E.W., Keeling A.G. (1998). Thick film tin oxide sensors for detecting carbon monoxide at room temperature. J. Mater. Sci.: Mater. Electron.

[8] Kim D.H., Lee S.H., Kim K.H. (2001). Comparison of CO-gas sensing characteristics between mono- and multi-layer Pt/SnO_2_ thin films. Sens. Actuat. B.

[9] Sauvan M., Pijolat C. (1999). Selectivity improvement of SnO2 films by superficial metallic films. Sens. Actuat. B.

[10] Tamaekong N., Liewhiran C., Wisitsoraat A., Phanichphant S. (2009). Sensing Characteristics of Flame-Spray-Made Pt/ZnO Thick Films as H_2_ Gas Sensor. Sensors.

[11] Safonova O.V., Delabouglise G., Chenevier B., Gaskov A.M., Labeau M. (2002). CO and NO_2_ gas sensitivity of nanocrystalline tin dioxide thin films doped with Pd, Ru and Rh. Mater. Sci. Eng. C.

[12] Safonova O.V., Rumyantseva M.N., Ryabova L.I., Labeau M., Delabouglise G., Gaskov A.M. (2001). Effect of combined Pd and Cu doping on microstructure, electrical and gas sensor properties of nanocrystalline tin dioxide. Mater. Sci. Eng. B.

[13] Zhang G., Liu M. (2000). Effect of particle size and dopant on properties of SnO_2_-based gas sensors. Sens. Actuat. B.

[14] Pagnier T., Boulova M., Galerie A., Gaskov A., Lucazeau G. (2000). Reactivity of SnO_2_-CuO nanocrystalline materials with H_2_S: A coupled electrical and Raman spectroscopic study. Sens. Actuat. B.

[15] Fukui K., Katsuki A. (2000). Improvement of humidity dependence in gas sensor based on SnO_2_. Sens. Actuat. B.

[16] Choi S.D., Lee D.D. (2001). CH_4_ sensing characteristics of K-, Ca-, Mg impregnated SnO_2_ sensors. Sens. Actuat. B.

[17] Ivanovskaya M., Bogdanov P., Faglia G., Nelli P., Sberveglieri G., Taroni A. (2001). On the role of catalytic additives in gas-sensitivity of SnO_2_-Mo based thin film sensors. Sens. Actuat. B.

[18] Han C.-H., Han S.-D., Khatkar S. (2006). Enhancement of H_2_-sensing properties of F-doped SnO_2_ sensorby surface modification with SiO_2_. Sensors.

[19] Kim I., Han S., Han C., Gwak J., Lee H., Wang J. (2006). Micro semiconductor CO sensors based on indium-doped tin dioxide nanocrystalline powders. Sensors.

[20] Kwon C.H., Yun D.H., Hong H.K., Kim S.R., Lee K., Lim H.Y., Yoon K.H. (2000). Multi-layered thick-film gas sensor array for selective sensing by catalytic filtering technology. Sens. Actuat. B.

[21] Hossein-Babaei F., Orvatinia M. (2003). Analysis of thickness dependence of the sensitivity in thin film resistive gas sensors. Sens. Actuat. B.

[22] Batzill M. (2006). Surface science studies of gas sensing materials: SnO_2_. Sens. J.

[23] Sakai G., Matsunaga N., Shimanoe K., Yamazoe N. (2001). Theory of gas-diffusion controlled sensitivity for thin film semiconductor gas sensor. Sens. Actuat. B.

[24] Hossein-Babaei F., Hosseini-Golgoo S.M., Amini A. (2009). Extracting discriminative information from the Padé-Z-transformed responses of a temperature-modulated chemoresistive sensor for gas recognition. Sens. Actuat. B.

[25] Vergara A., Martinelli E., Llobet E., Giannini F., D'Amico A., Di Natale C. (2007). An alternative global feature extraction of temperature modulated micro-hotplate gas sensors array using an energy vector approach. Sens. Actuat. B.

[26] Vergara A., Llobet E., Brezmes J., Ivanov P., Cané C., Gràcia I., Vilanova X., Correig X. (2007). Quantitative gas mixture analysis using temperature-modulated micro-hotplate gas sensors: Selection and validation of the optimal modulating frequencies. Sens. Actuat. B.

[27] Ding H., Ge H., Liu J. (2005). High performance of gas identification by wavelet transform-based fast feature extraction from temperature modulated semiconductor gas sensors. Sens. Actuat. B.

[28] Sysoev V., Kiselev I., Frietsch M., Goschnick J. (2004). Temperature gradient effect on gas discrimination power of a metal-oxide thin-film sensor microarray. Sensors.

[29] Hossein-Babaei F., Hosseini-Golgoo S.M. (2008). Analyzing the responses of a thermally modulated gas sensor using a linear system identification technique for gas diagnosis. IEEE Sens. J.

[30] Gutierrez-Osuna R., Gutierrez-Galvez A., Powar N. (2003). Transient response analysis for temperature-modulated chemoresistors. Sens. Actuat. B.

[31] Gutierrez-Osuna R., Nagle H.T. (1999). A method for evaluating data-preprocessing techniques for odor classification with an array of gas sensors. IEEE Trans. Syst. Man Cybern. B.

[32] Kermani B.G., Schiffman S.S., Troy Nagle H. (1999). Using neural networks and genetic algorithms to enhance performance in an electronic nose. IEEE Trans. Biomed. Eng.

[33] Phaisangittisagul E., Nagle H.T. (2007). Enhancing multiple classifier system performance for machine olfaction using odor-type signatures. Sens. Actuat. B.

[34] Phaisangittisagul E., Nagle H.T. (2008). Sensor selection for machine olfaction based on transient feature extraction. IEEE Trans. Insrum. Meas.

[35] Hossein-Babaei F., Orvatinia M. (2003). Gas diagnosis based on selective diffusion retardation in an air filled capillary. Sens. Actuat. B.

[36] Hossein-Babaei F., Hemmati M., Dehmobed M. (2005). Gas diagnosis by a quantitative assessment of the transient response of a capillary-attached gas sensor. Sens. Actuat. B.

[37] Bahraminejad B., Basri S., Hambali Z., Isa M. (2009). Single selective gas sensor for detecting flammable gases. IEICE Electron. Express.

[38] Gutierrez-Osuna R. (2002). Pattern analysis for machine olfaction: A review. IEEE Sens. J.

[39] Bahraminejad B., Basri S., Hambali Z., Isa M. (2010). Evaluation of dimension effects on capillary-attached gas sensor. Meas. Sci. Technol..

[40] Lee S.W., Tsai P.P., Chen H. (2000). Comparison study of SnO_2_ thin- and thick-film gas sensors. Sens. Actuat. B.

[41] El Khakani M.A., Dolbec R., Serventi A.M., Horrillo M.C., Trudeau M., Saint-Jacques R.G., Rickerby D.G., Sayago I. (2001). Pulsed laser deposition of nanostructured tin oxide films for gas sensing applications. Sens. Actuat. B.

[42] Gutierrez-Osuna R., Nagle H.T., Kermani B., Schiffman S.S., Pearce T.C., Schiffman S.S., Nagle H.T., Gardner J.W. (2002). Signal conditioning and pre-processing. Handbook of Machine Olfaction: Electronic Nose Technology.

[43] Hines E.L., Boilot P., Gardner J.W., Gongora M.A., Pearce T.C., Schiffman S.S., Nagle H.T., Gardner J.W. (2002). Pattern Analysis for Electronic Noses. Handbook of Machine Olfaction: Electronic Nose Technology.

[44] Distante C., Leo M., Siciliano P., Persaud K.C. (2002). On the study of feature extraction methods for an electronic nose. Sens. Actuat. B.

[45] Pardo M., Sberveglieri G. (2007). Comparing the performance of different features in sensor arrays. Sens. Actuat. B.

[46] Bellman R.E. (1961). Adaptive Control Processes: A Guided Tour.

[47] Bishop C.M. (1995). Neural Networks for Pattern Recognition.

[48] Duda R.O., Hart P.E., Stork D.G. (2000). Pattern Classification.

[49] Hastie T., Tibshirani R., Friedman J. (2001). The Elements of Statistical Learning: Data Mining, Inference, and Prediction.

[50] Pardo M., Sberveglieri G. (2005). Classification of electronic nose data with support vector machines. Sens. Actuat. B.

[51] Brudzewski K., Osowski S., Markiewicz T. (2004). Classification of milk by means of an electronic nose and SVM neural network. Sens. Actuat. B.

[52] Distante C., Ancona N., Siciliano P. (2003). Support vector machines for olfactory signals recognition. Sens. Actuat. B.

[53] Demuth H., Beale M. (2004). Neural Network Toolbox User’S Guide: For Use with Matlab, Version 4.

[54] Liang X., Xiaodong W. Gas quantitative analysis with support vector machine.

[55] Jain A.K., Duin R.P.W., Mao J. (2000). Statistical pattern recognition: A review. IEEE Trans. Patt. Anal. Mach. Intell.

